# Somatic Cell Reprogramming for Nervous System Diseases: Techniques, Mechanisms, Potential Applications, and Challenges

**DOI:** 10.3390/brainsci13030524

**Published:** 2023-03-22

**Authors:** Jiafeng Chen, Lijuan Huang, Yue Yang, Wei Xu, Qingchun Qin, Rongxing Qin, Xiaojun Liang, Xinyu Lai, Xiaoying Huang, Minshan Xie, Li Chen

**Affiliations:** 1 Department of Neurology, the First Affiliated Hospital of Guangxi Medical University, Nanning 530021, China; 2 Key Laboratory of Longevity and Aging-Related Diseases of Chinese Ministry of Education, Nanning 530021, China

**Keywords:** nervous system diseases, neuroscience, somatic cell, reprogramming, neurons, mechanisms, transcription factors, therapeutic, microRNA, molecules

## Abstract

Nervous system diseases present significant challenges to the neuroscience community due to ethical and practical constraints that limit access to appropriate research materials. Somatic cell reprogramming has been proposed as a novel way to obtain neurons. Various emerging techniques have been used to reprogram mature and differentiated cells into neurons. This review provides an overview of somatic cell reprogramming for neurological research and therapy, focusing on neural reprogramming and generating different neural cell types. We examine the mechanisms involved in reprogramming and the challenges that arise. We herein summarize cell reprogramming strategies to generate neurons, including transcription factors, small molecules, and microRNAs, with a focus on different types of cells.. While reprogramming somatic cells into neurons holds the potential for understanding neurological diseases and developing therapeutic applications, its limitations and risks must be carefully considered. Here, we highlight the potential benefits of somatic cell reprogramming for neurological disease research and therapy. This review contributes to the field by providing a comprehensive overview of the various techniques used to generate neurons by cellular reprogramming and discussing their potential applications.

## 1. Introduction

The world’s population is aging quickly, and this is linked to several systemic diseases such as neurological disorders [1]. Notably, the number of people with neurological diseases increases as the population ages [2]. As of 2019, in the elderly, stroke and Alzheimer’s disease are among the top 10 causes of death [3]. This growing patient population significantly burdens healthcare systems, society, and the economy [2]. Unfortunately, effective treatments for degenerating or damaged neurons have yet to be developed in neuroscience [4].

Obtaining neural tissue or neurons for research is often challenging due to limitations such as ethics or hardships in obtaining the tissue. The human brain and spinal cord are protected by the skull and spinal canal, and obtaining tissue often implies traumatic surgery. The lack of human in vitro models to investigate pathological alterations in neural cell function that affect the entire neurological disease process is a severe limitation in studying mechanisms and potential therapies to protect brain health and target age-related neurodegenerative diseases.

Finding new ways to study diseases and treat neurological disorders is crucial in this urgent situation. Somatic cell reprogramming, a new way to obtain neurons, is one way that scientists are trying to do this. Through somatic cell reprogramming, somatic cells can be transformed into neurons, thereby accelerating research progress and advancing the development of effective neural repair and replacement solutions [5]. Somatic reprogramming involves transforming one somatic cell type into induced pluripotent cells, which are then matured into the desired cell type. In contrast, transdifferentiation refers to the direct transformation of one somatic cell type into another without first reprogramming into pluripotent cells and then differentiating into functional somatic cells [6].

In general, mature neurons that are lost in adulthood are not replaced. The brain does contain some pockets of neural stem cells, including the subventricular zone across the lateral ventricles and the subgranular zone of the dentate gyrus in the hippocampus [7,8]. However, these neural stem cells are relatively sparse and cannot restore the large numbers of neurons lost during stroke, major trauma, or neurodegenerative disease [8]. Thus, scientists have focused on utilizing somatic cells to acquire neurons to address the scarcity of resources for studying human neurological diseases [9]. 

Early research on somatic cell reprogramming can be traced back to the middle of the last century, when scientists attempted to perform nucleus transplantation, and the transplanted cells were able to develop into intact individuals. For example, Gurdon et al. transferred a nucleus from an embryonic cell into an enucleated and unfertilized egg of the same species; this cell eventually developed into an entire sexually mature individual of *Xenopus laevis* [10]. Another example is Wilmut et al.’s method of cloning sheep by transplanting adult nuclei into unfertilized egg cells, a study known worldwide as the “birth of Dolly, the sheep” [11]. These two experiments showed that the nuclei of adult cells could be reprogrammed so that they exhibited cellular characteristics like those of fertilized eggs. In a later study, Japanese scientist Tomo Nakayama transplanted the nuclei of adult rat skin cells into embryonic stem cells. This study showed that the nuclei of adult cells could be reprogrammed into embryonic stem cells. These stem cells could differentiate into different types of cells [12]. 

Subsequent progress was that different reprogramming methods, such as transcription factors, were developed. In 2004, researchers dove into the mechanisms of nuclear reprogramming of adult cells. They found that specific transcription factors could reprogram somatic cells into pluripotent stem cells (capable of differentiating into many different types of cells). In 2006, scientist Shinya Yamanaka successfully transformed adult mouse skin cells into pluripotent stem cells capable of differentiating into multiple cell types by subjecting them to genetic transformations and cultures [13], which led to his winning the Nobel Prize. This achievement was considered a revolutionary advancement in cell biology and medicine and laid the foundation for subsequent research. In subsequent studies, scientists discovered that it was possible to obtain mature neurons by converting somatic cells into stem cells and then differentiating them into neural precursor cells with a dozen transcription factors [14,15,16,17].

Indeed, ectopic overexpression of specific neuronal determinants can reprogram non-neuronal cells directly into fully functional neurons in vivo and in vitro [18,19,20]. Since this pioneering work, researchers have explored how to exploit transcription factors, microRNAs (miRNAs), and gene silencing to reprogram highly differentiated, mature cells into neurons for therapeutic purposes. Generally, the rationale is to suppress the expression of genes subserving the current cell phenotype while activating genes that give rise to the target cell phenotype [21,22]. 

The complete reprogramming of somatic cells has evolved step by step, from early studies of nuclear transplantation of adult cells, to the discovery that pluripotency was induced in somatic cells overexpressing certain transcription factors, to the ability of somatic cells to transdifferentiate directly into neurons without going through the pluripotency stage. Sophisticated techniques and tools are now available to reprogram somatic cells into neurons. The next phase of research should be to dig deeper into the mechanisms of reprogramming, promote the efficiency gain of cell reprogramming, limit the risks of reprogramming, improve the safety of reprogramming in vivo, and, most importantly, develop therapeutic modalities that can be applied to clinical patients to truly bring the benefits of somatic cell reprogramming technology to patients with neurological diseases.

This review analyzes the history of efforts to reprogram adult somatic cells into neurons. First, we focus on somatic cell reprogramming and adult cell types that can be reprogrammed into neurons. We explore transdifferentiation strategies and factors and the molecular pathways involved. Subsequently, we explore the therapeutic potential of such somatic cell reprogramming as a treatment against neurological injury and illness and highlight the challenges that must be overcome. Researchers interested in reprogramming can use this review to obtain a quick overview of the technical means of reprogramming somatic cells into neurons and the related application studies. This review can also reveal future trends in somatic cell reprogramming into neurons.

## 2. Cell Types That Have Been Researched for Transdifferentiation into Neurons

Multiple types of somatic cells have been investigated in neural reprogramming. Reprogramming is easier and more effective when the somatic cell phenotype is similar to the target neuron lineage. Astrocytes are currently thought to be ideal candidates for neural repair. Human fibrocytes are also other ideal sources of reprogramming in this field because they are relatively easy to obtain.

Researchers have investigated the reprogramming of non-neuronal somatic cells into neurons using different cell types from other organs and embryonic cortices, with most studies focusing on ectodermal cell types. These ectodermal cells include fibroblasts [13,23,24,25], keratinocytes, oligodendrocytes [24], astrocytes [26], pericytes [27], and neuronal cells with the same neuronal identity [28]. T cells [29] and monocytes [30] from peripheral blood and hepatocytes [31] from visceral organs can also be reprogrammed into neurons. The success of these attempts has provided multiple cellular resources for neural reprogramming.

The more similar the initial adult cell phenotype is to the target neuronal phenotype, the more straightforward and effective the transdifferentiation procedure is. This is because embryonic stem and progenitor cells can more easily differentiate into cells with similar genealogical origins. The neuronal ectoderm produces fibroblasts, astrocytes, and pericytes [32]. Most early studies tried to generate neurons from fibroblasts, but astrocytes may be the ideal candidates for neuroregenerative reprogramming [33]. The main cell types used for brain regeneration and reprogramming are shown in Table 1.

The somatic cell types presented in the table above are the somatic cells that are currently used for reprogramming into neurons. Some of these cells, such as fibroblasts and astrocytes, are used more frequently. Despite their abundance, most types of cells are not formed into sustainable cell lines, and the need to continuously isolate cultures from the body’s limited tissues is a significant limitation.

## 3. Mature Somatic Cells Reprogram into Neurons through Different Pathways

Somatic cell reprogramming into neurons can occur by inducing pluripotency, stimulating neural stem cells, or directly reprogramming cells into specific neuronal subtypes. Induced pluripotent stem cells (iPSCs) can differentiate into different types of neurons, whereas neural stem cells only differentiate in specific environments. Adult neurons can be generated using direct reprogramming without the use of pluripotent or neural stem cells.

First, somatic cells can be forced to become pluripotent. Then, these stem cells differentiate into different kinds of neurons. For example, induced pluripotent stem cells (iPSCs) such as peripheral blood mononuclear cells display pluripotency after transdifferentiation; they can be converted into motor neurons using differentiation medium containing brain-derived neurotrophic factor (BDNF) or glial cell line-derived neurotrophic factor (GDNF) [30,54]. The resulting neurons express mature neuronal markers such as Tuj1, Map2, and synaptophysin, and they show spontaneous action potentials in patch clamp assays [14,15,16,17]. 

Second, somatic cells are stimulated to produce neural stem cells, which then differentiate into neurons in a particular environment, such as in differentiation medium. For example, treating oligodendrocyte precursors with bone morphogenetic protein generates neural stem cells that express stem cell markers and that can be incubated with exogenous factors to differentiate into oligodendrocytes, astrocytes, and neurons [51,55,56,57].

**Figure 1:**
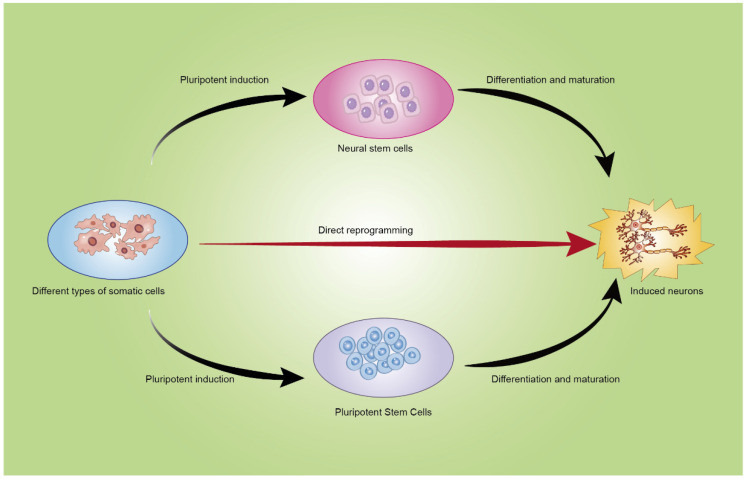
Pathways of neural reprogramming. First, some cells can be treated with induction factors to become pluripotent stem cells or neural stem cells, which then mature into neurons. Some somatic cells can differentiate directly into neurons.

Third, somatic cells do not undergo the intermediate step of pluripotent stem cells or neural stem cells but direct reprogramming to specific neuronal subtypes. Overexpressing proneural transcription factors in astrocytes or applying exogenous factors can generate adult neurons without the need to induce pluripotent or neural stem cells [58,59,60,61,62,63]. During direct reprogramming, cells enter a transient pluripotent stage in which several neurogenesis-related genes are upregulated to complete the conversion into neurons [64]. Figure 1 depicts how induction can change non-neurons or neurons into target neurons.

Above all, somatic cells can be reprogrammed into neurons in three different ways. The reprogramming pathway differs for different types of cells, based primarily on the cells’ characteristics and the method of induction used. The more general approach of direct reprogramming without going through the induced pluripotent stem cell phase may reduce the risk of tumorigenesis.

## 4. Transcription Factors, Small Molecules, and miRNAs That Induce Transdifferentiation of Somatic Cells into Neurons

Scientists have used different methods to convert somatic cells into neurons, such as transcription factors, small chemical molecules, and microRNAs. Both transcription factors and microRNAs are essential in early neural development. The small molecules are generally involved in critical cellular pathways, and in turn, all three can be reshaped into somatic cell phenotypes, toward a neuronal phenotype.

The observation that bone morphogenetic factors can stimulate oligodendrocyte progenitors to differentiate into oligodendrocytes, astrocytes, and neurons [57] prompted researchers to examine new techniques for transforming somatic cells into neurons by using transcription factors, small molecules, and miRNAs (Table 2 and Table 3).

Embryonic nervous system development involves numerous proneural transcription factors, including Ascl1, Mash1, Neurog1–3, Math1, KLF4, MYC, POU5F1, NeuroD1, Pax6, and Sox2. Ascl1 and Neurog2 induce growing stem cells to become an intermediate neuronal subtype [65]. Ascl1 regulates progenitor maintenance, neuronal differentiation, and neurite development in the central and peripheral nervous systems [66]. Based on their critical role in neurodevelopment, Ascl1 and Neurog2 cause somatic reprogramming by ectopic overexpression in somatic cells [48,49,67]. Pax6 is a member of the paired-box transcription factor family widely expressed in the developing central nervous system [68], where it regulates cortical progenitor cell proliferation, neurogenesis, migration, and forebrain axonal connections [69]. It also guides the differentiation of glial cells into neurons during embryonic mouse brain formation. Table 2 illustrates transcription factor families and their reprogramming roles.

**Table 2:** Transcription factors commonly used for reprogramming somatic cells into neurons.

Transcription Factor	Family Affiliation	Role in Neurogenesis, Differentiation, or Reprogramming	References
ASCL1	bHLH family	Determination of neuronal subtypes during neural development.	[70]
ATOH1	bHLH family	Unknown.	[71]
BCL11B	COUP TF1-interacting protein 2 (also known as Ctip2) and zinc finger-containing transcriptional repressors	Central to differentiation of medium spiny neurons and development of the striatum.	[72]
BCL2	Anti-apoptotic factor	Promotes DNA damage, genetic instability, and cell proliferation.	[33,73,74]
BRCA1	Tumor suppressor protein	Involved in BMP-2-mediated reactivation of Sox2.	[51]
BRN2a	Brain-specific homeobox/POU domain protein 2	Associated with neuroendocrine function.	[6,75,76]
CEND1	Neurogenic protein	Pathways involved in neuronal differentiation by CEND1 through activation by NEUROGENIN 1 and 2.	[77]
EBF1	Zinc finger	Acting downstream of Ngn, EBF-1 can promote ectopic neurogenesis.	[78,79]
FEZF2	Zinc finger transcriptional repressor	Fezf2 manipulates the origin, specific differentiation, and synaptic connectivity of corticospinal motor neurons by regulating neural progenitor cell lineage-directed differentiation signals.	[80]
FOXA2	Forkhead	Expressed in the ventral hindbrain’s serotonergic progenitor regions and in midbrain dopaminergic neurons.	[28,81]
FOXG1	Forkhead	Involved in the primitive (anterior) neuroectoderm during development of embryonic stem cells.	[82,83]
GATA3	Zinc finger	Associated with noradrenergic phenotype and development of the sympathetic nervous system.	[84,85]
GATA4	GATA	GATA4 can drive embryonic Sertoli-like cell differentiation.	[86]
HAND2	bHLH family	Required for the acquisition of noradrenergic phenotype.	[84,85,87]
ISL2	LIM homeodomain-containing	Vital to the development and differentiation of visceral motor neurons in the spinal cord.	[78,88]
KLF4	Zinc finger	Directly represses p53.	[13]
LIN28	RNA binding protein	Lin-28 can shuttle between the nucleus and cytoplasm and regulate other genes that control the cell cycle.	[89]
LMO2	Key hematopoietic transcriptional regulator	Creates a regulatory complex that mediates transcription of multiple genes in hematopoietic progenitor cells; it is associated with the transcriptional control of stem/progenitor cells.	[90,91]
c-Myc	Myc	Alters expression of many proteins to enhance proliferation and transformation.	[13]
MYT1L	Neural zinc finger	Exemplifies a class of neural sequence-specific transcription factors that actively recruit histone deacetylases to selected genes during central nervous system development.	[92,93]
NANOG	Divergent homeodomain protein	NANOG sustains the identity of embryonic stem cells (ESCs).	[94]
NEUROD1	bHLH family	Neural differentiation factor essential for late-stage neurogenesis and important in the development of the central nervous system, as well as in the auditory and vestibular systems.	[35,95,96]
NEUROD2	bHLH family	Essential for the maturation and survival of neurons in the central nervous system.	[97,98,99]
NEUROG2	bHLH family	NEUROG2 is a key contributor to early neurogenesis.	[67]
NURR1	Nr4a2 (ligand-independent nuclear receptors)	Essential for the differentiation, maturation, and maintenance of midbrain dopaminergic neurons.	[100,101]
OCT/4	POU5F1, a member of the POU class of homeodomain proteins	Central to the transcriptional regulatory hierarchy that specifies embryonic stem cell identity during early development.	[102]
OLIG2	Basic helix–loop–helix	Mediates self-renewal in the expansion of neurosphere cultures and promotes the generation of neurons and oligodendrocytes under differentiation conditions.	[103]
PAX6	Paired-box family	Critically important in multiple cell types and at several stages of forebrain development.	[104]
PHOX2A	Paired homeodomain	Selectively expressed and required for the specification of ventral motor neurons in the hindbrain and in the oculomotor nucleus, located laterally to dopaminergic neurons in the ventral midbrain.	[105]
Phox2B	Paired homeodomain	Same as Phox2a.	[105]
PTF1A	Basic helix–loop–helix	Mostly expressed in post-mitotic cells, and it specifies terminal cell fate in neural tissues.	[106]
SOX2	HMG-box	Central to the transcriptional regulatory hierarchy that specifies embryonic stem cell identity during early development.	[102]
SOX4	SoxC	Controls the survival of neural precursors and their differentiated progeny, in redundancy with SOX11.	[107]
SOX11	SoxC	Same as SOX4.	[107]
SV40LT	SV40 large T gene	Unknown.	[108]
TLX3	Tlx-class homeobox genes	Tlx3 functions as a post-mitotic selection gene in the embryonic spinal cord, determining the fate of dorsal glutamatergic neuronal cells.	[78,109]
ZEB1	Zinc finger E-box-binding transcription factor	During individual development, Zeb1 plays a crucial role in the nervous system. It is upregulated in growing neurons throughout the central nervous system and is required for the survival of spinal cord neural stem cells.	[110]

The basic helix–loop–helix protein family of transcription factors includes “proneuronal factors” [111], which play an indispensable role in neuronal commitment and in identifying neural progenitors. One such proneuronal factor is neurogenin2 (Ngn2), which controls neuronal development and identity [112]. Ngn2 regulates differentiation into the glutamatergic neuron phenotype, preventing the formation of γ-aminobutyric acid (GABA)-ergic neurons [70]. Ngn2 reprograms reactive astrocytes into deep cortical vertebral neurons that extend to the striatum, thalamus, and spinal cord [33,113,114].

Some of these transcription factors, alone or in combination, can induce non-neuronal somatic cells to reprogram into neurons. In a mouse model of cerebral infarction, overexpressed Olig2 and Pax6 reprogrammed glial cells into neurons in situ, as confirmed by the expression of the neuron-specific marker doublecortin and electrophysiological assays [115]. In another study, exogenous Brn2a, Myt1l, and ASCL1 induced mouse embryonic fibroblasts to differentiate into neurons that expressed microtubulin III (TUJ1) and microtubule-associated protein 2 (MAP2) [75]. POU5F1, SOX2, KLF4, and MYC can stimulate the development of non-neuronal somatic cells into neurons. One study overexpressed the transcription factors Oct4, Sox2, Klf4, and c-Myc in murine fibroblasts to obtain functional neural progenitor cells, which could differentiate into neurons and glial cells [116].

Hair follicle keratinocytes have been reprogrammed into iPSCs, which differentiate into neural precursors and, subsequently, into dopaminergic or glutamatergic neurons [32]. Overexpression of OCT3/4, Sox2, Klf4, and Myc in astrocytes led to iPSCs that expressed stem cell markers and showed potential to differentiate into neurons [117]. These various transcription factors can work as a complex or alone to reprogram non-neuronal mature somatic cells into neurons [49,62,118].

MicroRNAs also play an important role in the reprogramming of somatic cells into neurons. MicroRNAs (miRNAs) are key regulators of gene expression that control numerous cellular and developmental processes in eukaryotes [119]. Within the central nervous system (CNS), miRNAs play a critical role in the regulation of gene expression patterns during development [120], and are actively involved in the regulation of neurogenesis at each stage [121]. Moreover, miRNAs play a fundamental role in the establishment of specific neuronal phenotypes [122], such as miR-124, which is capable of inducing a neuronal phenotype when overexpressed in embryonic stem cells [123]. Embryonic stem cells are enriched in miRNA-124, miR-9/9*, and microRNAs 302-367, which can suppress the neural-gene-specific repressor REST complex, which is essential for the expression of genes related to neuronal function [26,98]. 

Researchers have successfully induced the reprogramming of fibroblasts into neurons using miR-9/9* and miR-124 [72,124], and active astrocytes into neurons in normal and Alzheimer’s disease models using microRNA-302/367 [26]. However, microRNA-302/367 acts as a mediator and enhances the induction effect of miR-9/9* and miR-124 on fibroblasts, and may not be sufficient by itself for fibroblast reprogramming [25]. Furthermore, miRNAs can be used in combination with other methods, such as the combination of miR-9/9* and miR-124 with transcription factors to induce the reprogramming of fibroblasts from bipolar disorder patients into neurons [97]. Additionally, miR-124-9-9* has been shown to increase the efficiency of Ascl1-induced reprogramming of Müller glia into neurons [125]. Taken together, miRNAs are important players in the reprogramming of somatic cells into neurons, providing exciting prospects for regenerative medicine research.

Dozens of small compounds have been used as activators or inhibitors of critical signaling pathways to regulate reprogramming, preserve cellular stability during transdifferentiation, and avoid cell death (Table 3). In 2013, a study revealed that exogenous small molecules were sufficient to convert mouse somatic cells into pluripotent stem cells [126]. Since then, various research teams have explored hundreds of small molecules for somatic cell reprogramming, including valproic acid, CHIR99021, repsox, forskolin, SP600125, GO6983, and Y-27632 [54]. Combinations of small molecules such as these can induce the transdifferentiation of fibroblasts, astrocytes, and glioblastomas into neurons [54,127,128].

**Table 3:** List of small molecules commonly used for reprogramming somatic cells into neurons and differentiating them during their maturation.

Small Molecule	Description	References
616452	Repsox, an ALK5 inhibitor.	[129]
17-allylaminogeldanamycin	GSK3 inhibitor.	[129]
A83-01	ALK4/5/7 inhibitor.	[38,130,131]
All-trans retinoic acid	Physiologically active metabolite of vitamin A.	[53,132,133]
AM580	Retinoic acid agonist, stable benzoic derivative of retinoic acid.	[129,134]
Apicidin	HDAC inhibitor.	[51]
Azacytidine	Nucleic acid synthesis inhibitor.	[135]
Blebbistatin	NMII inhibitor.	[136]
Bradykinin	Bradykinin plays a role in neural fate determination and facilitates neurogenesis and migration.	[137]
CH55	Synthetic stable analog of retinoic acid.	[138]
CHIR99021	GSK3 inhibitor.	[129]
CpdE	Notch signaling pathway inhibitor.	[139]
DAPT	Inhibits γ-secretase and Notch signaling.	[53,140]
DMH1	BMP type I receptor inhibitor.	[139]
Dorsomorphin	Inhibitor of AMP-activated protein kinase and bone morphogenetic protein type 1 receptor.	[67]
DZNep	Histone methylation inhibitor.	[141]
EPZ004777	Dot1l inhibitor.	[138,142]
Forskolin	cAMP agonist.	[129]
GSK3β inhibitor	Glycogen synthase kinase-3β boosts the production of neuroprotective and neurotrophic factors in the context of spinal cord injury.	[143]
GO6983	PKC inhibitor.	[54,144]
Hh-Ag1.5	Unknown.	[131]
I-BET 151	BET family bromodomain inhibitor.	[145]
Insulin–transferrin–selenium	Insulin, transferring, and sodium selenium compound.	[135]
Isoxazole	Isoxazole is able to upregulate proneural marker genes and exhibit regulation of stem cells.	[41]
ISX9	Induces neuronal differentiation through myocyte enhancer factor 2 (Mef2), which is a vital pathway for neural differentiation and maturation.	[128]
Kenpaullone	GSK-3β inhibitor.	[146]
LDN193189	Inhibitor of bone morphogenetic protein type I receptors ALK2 and ALK3, used to suppress specification of mesoderm and endoderm.	[147]
LIF	Leukemia inhibitory factor.	[148,149]
LM-22A4	Growth factor.	[150]
Mercaptoethanol	Unknown.	[148]
MS-275	Benzamide.	[51]
Niclosamide	Wnt signaling inhibitor.	[34]
Noggin	SMAD inhibitor.	[151]
NT3	Unknown.	[150]
P7C3-A20	May stimulate NAMPT-relevant pathways to exert neurogenesis.	[24]
Parnate	Lysine-specific demethylase 1 inhibitor.	[24]
PD0325901	Mitogen-activated protein kinase inhibitor.	[24]
PS48	PDK1 activator.	[152]
Purmorphamine	Activator of the Shh signaling pathway.	[153]
Quercetin	PI3K signaling inhibitor.	[34]
QVD-OPH	Caspase inhibitor.	[144]
Repsox	Transforming growth factor-β inhibitor.	[154]
Retinoic acid	Induces neurogenesis and neuronal differentiation by activating retinoic acid receptors.	[38]
RG108	DNA methyltransferase inhibitor that is less toxic to cells than parnate.	[24]
Ruxolitinib	Selective JAK1/2 inhibitor.	[155]
SB203580	P38 MARK inhibitor.	[145,155]
SB43152	Unknown.	[153]
SB431542	Inhibits TGF-β type I receptors ALK4, ALK5, and ALK7.	[30,147]
SB4352	Transforming growth factor-beta inhibitor	[36]
SMER28	SMER28 shows neurotrophic and neuroprotective effects by inducing neurite growth and protecting against excitotoxin-induced axonal degeneration.	[156,157]
Smoothened agonist	Alone or in concert with other molecules, smoothened agonist stimulates proliferation of primary neuronal precursor cells.	[158,159]
Sodium butyrate	HDAC inhibitor, causes hyperacetylation of histones.	[38]
Sonic hedgehog (SHH)	Required for the development of dopaminergic neurons in multiple locations along the anterior neural tube.	[160,161]
SP600125	1,9-pyrazoloanthrone, JNK inhibitor.	[162]
SP600625	JNK inhibitor.	[144]
TD114-2	GSK3-beta inhibitor, preferred over CHIR99021.	[138]
Transforming growth factor beta 3	Required for the induction, differentiation, and survival of midbrain dopaminergic neurons.	[163]
Thiazovivin	Unknown.	[164]
Tranylcypromine	Lysine-specific histone demethylase LSD1 inhibitor.	[141]
Trichostatin A	Histone deacetylase inhibitor.	[165]
TTNPB	Agonist of retinoic acid receptors, which play an important role in neural differentiation.	[164]
Valproic acid	Inhibits histone deacetylase activity.	[166]
Vitamin C	Prevents cell death.	[144]
Y-27632	Rho-associated protein kinase inhibitor.	[162]

Abbreviations: ALK: Anaplastic lymphoma kinase; GSK: glycogen synthase kinase; NMII: Nonmuscle myosin II; BMP: Bone morphogenetic protein; AMP: Adenosine monophosphate; Dot1l: Known as KMT4L; cAMP: Cyclic adenosine monophosphate; PKC: Protein Kinase C; BET family: Bromodomain and extra-terminal domain family; SMAD: small mother against decapentaplegic; NAMPT: Nicotinamide phosphoribosyltransferase; PDK1: 3-phosphoinositide-dependent kinase 1; PI3K: Phosphoinositide 3-kinase; JAK1/2: Janus kinases 1/2; MARK: Microtubule-affinity regulating kinases; TGF: Transforming growth factor; HDAC: Histone deacetylase; JNK: c-Jun N-terminal kinase; LSD1: Lysine-specific histone demethylase 1.

Transcription factors, microRNAs, and small molecules can be used individually or in combination to induce cellular reprogramming. When used together, they complement each other and increase efficiency of the reprogramming process. This is partly due to the ability of transcription factors to bind microRNAs [72] and small molecules [67]. For example, cells can undergo early stages of reprogramming by overexpressing transcription factors or adding microRNAs. The reprogrammed cells can then be induced to mature through the addition of valproic acid, forskolin, vitamin C, and BDNF [16,30]. Small molecules can also enhance reprogramming by modifying DNA or histone structure [164]. 

We have listed the technical approaches to reprogram somatic cells into neurons above, but these induction factors still need to be ideal. What we require is a method that is simpler, more efficient, safe, and clinically applicable. The current approach may still be in its infancy.

## 5. Molecular Mechanisms of Somatic Cell Transdifferentiation into Neurons

Cells are significantly transformed in all aspects of the reprogramming process, including changes in DNA plasticity, transcriptome, and energy usage. DNA plasticity refers to how inducing factors cause chromatin and gene expression to change over time. During reprogramming, there is also a dramatic change in the transcriptome. Genes related to the original somatic cell phenotype are downregulated, while genes related to the neural type are upregulated. Moreover, the energy process changes from glycolysis to aerobic oxidation, in which an induced neuron can exercise normal neurological function. A deeper understanding of these mechanisms can help us improve the efficiency of somatic reprogramming and prevent potential harm to the cells. A growing body of literature has explored the roles of changes in chromatin, transcription, translation, and metabolism in somatic reprogramming. Analysis of cell-specific markers and phenotypes has revealed three levels of change that may explain cellular transdifferentiation [67]. 

The first level is DNA plasticity. When cells are adequately triggered, such as in microglia overexpressing the transcription factor NeuroD1, DNA is dynamic [167], with changes in the accessibility of chromatin [42]. The expression of genes linked to neurons is upregulated, while motifs linked to the initial somatic cell phenotype are downregulated, as revealed by extensive RNA sequencing [147,168].

Even in mature, highly differentiated somatic cells, chromatin is not immutable, as demonstrated by the cloning of Dolly the sheep, macaques, and salamanders [10,169,170]. Mature somatic cell chromatin can be activated and altered to express specific genes. When somatic cells are reprogrammed, chromatin undergoes DNA methylation [171] and demethylation [172], which upregulates neurogenesis-related gene expression. One study found that demethylation of H3K9me3 in chromatin improved somatic cell nuclear transfer and promoted cellular reprogramming in mice and humans [173]. Direct fibroblast-to-neuron reprogramming involves DNA methylation remodeling; changes in DNA methylation inhibit fibroblasts’ myogenic program and promote activation of neuronal genes [171]. Genetic epistasis studies with H3K9 methyltransferase suggest that this chromatin change restricts plasticity by acting downstream of the end selector. Terminal selectors activate identity-specific genes and reduce the accessibility of non-identity-defining genes, balancing identity specification with cellular plasticity [174]. 

Secondly, emerging technologies such as RNA and single-cell sequencing support the idea that somatic cell reprogramming alters the transcriptome, such as in the case of glial cells [147] and fibroblasts [175]. Overall, genes related to maintaining the original somatic phenotype are downregulated during cell reprogramming, while genes associated with pluripotent stem cells, neuronal progenitors, or neuronal phenotypes are upregulated. For example, ASCL1 expression alters Müller cell chromatin and temporarily activates progenitor gene expression while suppressing glial cell gene expression, inducing Müller glia-derived progenitor cells to become neurons [49]. Ectopic expression of the neuronal transcription factor NeuroD1 causes rapid transcriptome changes during the astrocyte-to-neuron transition [176]. The shift toward a neuronal identity has been linked to changes in signaling pathways involving Notch and p21/p53 pathways [147,177]; indeed, inhibition of p53 or p21 increases production of induced adult neuroblasts from glial cells [177].

Third, during transdifferentiation, energy metabolism switches from glycolysis to aerobic oxidation. Other somatic cells, such as astrocytes, fibroblasts, brain stem cells, or progenitor cells, depend more on β-oxidation and anaerobic glycolysis for energy than neurons, which depend primarily on oxidative metabolism [178,179]. Furthermore, neuritogenesis can be enhanced by increasing the mitochondrial membrane potential, polarizing the mitochondria, and decreasing the reliance on glycolysis [180]. Because ATP generation is required for synaptic activity, neuronal activity strongly depends on normal functioning of neuronal mitochondria and energy metabolism [181,182]. As a result, one of the most visible aspects of the transdifferentiation process is the abrupt change in metabolism [33]. Cells surviving the severe energy change during reprogramming are candidates for becoming neurons [33]. At the same time, a dramatic energy shift and a significant accumulation of reactive oxygen species can harm cells and cause ferroptosis [33]. Therefore, a more in-depth study of metabolic shifts in successfully reprogramed somatic cells and exploration of ways to promote successful energy conversion and inhibit ferroptosis can improve reprogramming efficiency [33].

The central mechanism for reprogramming somatic cells into neurons is a phenotypic shift involving intracellular changes that should, in theory, be extensive. In addition to what we have shown above, other unknown mechanisms require more profound and advanced exploration methods.

## 6. Somatic Cell Transdifferentiation Provides New Possibilities for Research and Treatment of Neurological Disease

By reprogramming the somatic cells involved in neurological diseases, scientists can learn more about diseases and develop new treatment methods. This entails transforming somatic cells from patients with various genetic or age-related neurological diseases into neurons that retain the patient’s chromosomal mutations and display disease symptoms. In vitro disease modeling with induced neurons assists researchers in determining disease causes. In addition, researchers are investigating the possibility of reprogramming to reconstruct damaged neural networks and enhance the neural function of animals. Transforming induced neural stem cells into the mouse brain improved axonal regeneration, motor function, and electrophysiological activity. It has significant implications for the treatment of numerous diseases.

Somatic cell reprogramming for neurological disease research allows access to elusive tissues, facilitating investigations into disease mechanisms and novel interventions. For instance, somatic cells procured from patients afflicted with a range of genetic and age-related neurological disorders can be induced to differentiate into neurons. These induced neurons, which retain the patient’s chromosomal mutations [183] or display characteristics such as epilepsy-like hyperactivity [184] and defective neural networks in patients with autism [185,186], enable investigations into the underlying mechanisms of the diseases. Furthermore, motor neurons induced directly from fibroblasts taken from elderly patients exhibit age-related characteristics [187], making them an exceptional model for investigating late-stage motor neuron diseases. In vitro modeling is valuable for examining disease mechanisms, drug screening, and toxicity testing in patients with neurological disorders.

The induction of neurons holds potential for the treatment of neurological diseases. Research teams are using methods such as cell transplantation or in vivo reprogramming to reconstruct damaged neural networks and improve animal neural function. Corti et al. transplanted neural stem cells obtained from in vitro reprogramming of somatic cells into the mouse brain, which successfully integrated into the local brain cortex [94]. The transplanted induced neural stem cells could differentiate into all neural lineages and restore axonal regeneration in a spinal cord injury (SCI) model [188]. It was discovered that iNSC transplantation could promote motor function and electrophysiological activity recovery, as confirmed by functional evaluation. The use of somatic cell reprogramming has significant implications for treating a range of diseases, including neurodegenerative diseases, neurological disorders, and psychiatric conditions [18,20,35,95]. We have summarized the research on somatic cell reprogramming to become neurons for neurodegenerative diseases, neurological diseases, and psychiatric diseases in Table 4.

The reprogramming of somatic cells into neurons holds significant potential for the treatment of neurological diseases, with focus on disease processes and therapeutic applications. This approach can advance our understanding of disease development and and facilitate relevant drug screening and testing. There will still be gaps in treatment due to unresolved risks and safety issues. As a result, treatment is still in the animal model stage.

## 7. Limitations of Neural Reprogramming

Reprogramming somatic cells into neurons has the potential to be beneficial. However, limitations and risks cannot be overlooked, such as low efficiency, immune rejection, genetic mutations, tumorigenicity, and potentially unknown risks. The reprogramming process varies in efficiency, with reported rates ranging from as low as 0.01% [193] to as high as 90% [199]. The attainment of sufficient neuron numbers requires careful consideration of multiple factors, including cell type and reprogramming technique. Another limitation and danger of somatic cell reprogramming is the potential for immunological rejection, as reprogrammed cells may be viewed as foreign by the immune system. Even induced pluripotent stem cells (iPSCs) derived from host mice can elicit immunological rejection and cause teratomas when transplanted into animals [200]. The immunogenicity of iPSCs is attributed to genetic and epigenetic defects, which warrants caution.

The long-term stability of generated neurons is also a concern, as cells must maintain normal physiological function after reprogramming. However, concerns remain about the quality and durability of generated neurons, limiting the potential of somatic cell reprogramming.

Safety is the most critical issue in somatic cell reprogramming due to the potential risks. During reprogramming, genetic mutations may occur as a result of random integration of viral gene segments carried by retroviruses or lentiviruses into host cells, leading to alterations of essential host cell genes that may cause deleterious consequences or even cell death [201,202,203]. Uncontrolled proliferation and differentiation during reprogramming may result in tumor growth, while unanticipated genomic changes during gene editing can lead to the formation of malignant tumors [204]. The retroviruses used by Yamanaka carry a potential cancer risk, as they can fuse with the DNA of host cells. Furthermore, the Myc gene, one of Yamanaka’s four reprogramming factors, is an oncogene [205,206].

The safety risks associated with therapeutic somatic cell reprogramming persist, requiring further research to bridge the gap between laboratory research and clinical application. Improving the safety and reliability of the technology should be a primary focus of future research to protect human health while facilitating practical use. Given the significant obstacles, it is clear that there are substantial hurdles to overcome before this approach can be employed at the bedside.

## 8. Discussion

Reprogramming somatic cells into neurons has emerged as a prominent field of research over the past 20 years. This breakthrough has not only highlighted the plasticity of somatic cells but also opened up new possibilities for research and therapy in regenerative medicine. A wide range of somatic cells, including fibroblasts [24], astrocytes [26], pericytes [27], peripheral blood mononuclear cells [30], T lymphocytes [29], hepatocytes [31], and other cells, can now be transformed into neurons. With the refinement and standardization of techniques, the means to achieve this transformation are becoming more sophisticated and standardized. Several factors, such as transcription factors [13], microRNA [72,124], and a combination of different small chemical molecules [54], have been shown to induce the reprogramming of somatic cells into neurons. These methods, when combined, can further enhance reprogramming efficiency and stabilize induced neuronal cell stability.

During the reprogramming process, some somatic cells reprogrammed into neurons may undergo cellular pluripotency, reversing their differentiation from somatic cells to induced pluripotent stem cells or neural stem cells, followed by induced differentiation and maturation. This process gradually allows induced pluripotent stem cells or neural stem cells to differentiate and mature, acquiring neuron-associated phenotypes, neuron-like morphology, electrophysiological functions, and neuron-associated markers [51,57]. On the other hand, some somatic cells do not undergo pluripotency in response to inducing factors, instead undergoing direct reprogramming into another phenotypically distinct neuronal cell [64]. This process is also known as transdifferentiation. The reprogramming of somatic cells into neurons is a promising area of research that has demonstrated the potential of somatic cells in regenerative medicine. With the refinement and standardization of reprogramming techniques, this field holds great promise for future advances in the field of neuroscience research and regenerative medicine.

Many studies have primarily focused on the reprogramming of adult cells into neurons, yet the physiological and biochemical mechanisms involved in this process remain largely unknown. In this review, we aimed to integrate several studies in the literature and to provide a comprehensive summary of the mechanisms involved in reprogramming at three levels of physiological and biochemical processes. Firstly, at the chromatin level, the reprogramming process involves changes to the epigenetic landscape of somatic cells. These alterations affect the accessibility of reprogramming techniques [67], leading to changes in gene expression patterns. Secondly, the overall transcriptome level of somatic cells shifts towards a neuronal identity [204], characterized by the upregulation of genes associated with the neuronal phenotype and the downregulation of genes related to the original cell phenotype. Finally, at the metabolic level, the reprogramming process requires significant changes to cellular metabolism to meet the high energy demands of neuronal cells [33]. The reprogramming of somatic cells into neurons is a complex process that involves physiological and biochemical processes at multiple levels. Our review sheds light on the fundamental mechanisms underlying this process and may pave the way for future advancements in regenerative medicine and neuroscience.

The technique of reprogramming somatic cells into neurons is now a well-established and feasible method. This process allows us to have a more sustainable source of research material for studying neurological diseases. Moreover, several studies have been conducted on the in vitro reprogramming of neurons to establish disease models for various neurological disorders, such as ALS [181]. In animal models, cell transplantation therapy has been shown to effectively restore damaged neurological function [207].

While the field of somatic cell reprogramming for neurons is rapidly advancing, it is essential to acknowledge that this process also poses risks and challenges. Issues such as tumor formation, immunological rejection, cellular stability, the highly variable efficiency of reprogramming, and the ability of reprogrammed neuronal cells to integrate into local networks and function correctly are all critical factors that require our attention [5].

As previously mentioned, reprogramming adult cells into neurons involves the overexpression of a neural transcription factor in adult cells using a viral vector carrying the target gene, which is then expressed after viral infection of the host cell. The target gene is integrated into the chromatin of the host cell, and random mutations in the chromatin of the somatic cell can occur [201,202,203]. Sometimes, the cell may even die outright if the mutation occurs at a binding site. The induction of chemical molecules in a manner that does not involve gene insertion can also have toxic effects [37].

Reducing the risks and concerns associated with somatic cell reprogramming is necessary. To achieve this, it is essential to continue to improve and standardize the reprogramming process to make it as safe and efficient as possible. Additionally, further research is needed to better understand the underlying mechanisms of reprogramming and the factors that influence its effectiveness.

While reprogramming somatic cells into neurons holds great promise for advancing our understanding and treatment of neurological diseases, we must remain vigilant of the potential risks and challenges associated with this technique. By continuing to refine our methods and understanding the underlying mechanisms involved, we can ensure this technology’s safe and practical application in the clinic. Therefore, non-integrating approaches for somatic cell reprogramming have been proposed to address these issues. Various methods have been suggested, such as using a plasmid to construct the overexpressed gene and introducing it into the somatic cell via electroporation to achieve overexpression. Gotz’s research team has made significant progress in somatic cell reprogramming by using the CRISPR-Cas9 system to facilitate gene overexpression while avoiding the introduction of random mutations during cell reprogramming [208].

Furthermore, inducing pluripotency in somatic cells before differentiation into neurons may lead to the overexpression of genes associated with tumor formation and uncontrolled cellular proliferation. Additionally, the transcription factor c-MYC, commonly used in pluripotency induction, is inherently tumorigenic and closely linked to tumor formation and invasion [205,206]. Hence, it is essential to carefully select reprogramming factors to ensure successful and efficient induction and develop more advanced monitoring and control tools to establish the basis for future clinical applications of somatic reprogramming. In addition, further animal models and mechanistic studies are necessary to apply this tool in the clinical setting. Several research teams are currently using non-human primates to establish studies related to somatic reprogramming [209]. Non-human primates are the animals closest to humans regarding physiology, biochemistry, and metabolism, and strengthening animal studies is necessary to translate research into the clinic.

Reprogramming somatic cells into neurons has opened up an innovative and promising avenue for developing cell-based therapies for neurological diseases. This method could overcome the limitations of conventional treatments and provide a more effective and targeted approach to treating neurological disorders. Furthermore, the ability to generate patient-specific neurons through reprogramming could improve our understanding of the underlying mechanisms of neurological diseases and facilitate the development of personalized treatments. Reprogramming somatic cells into neurons is valuable for investigating and treating neurological diseases. With continued advancements in cellular reprogramming, we hope for significant progress in developing novel treatments and therapies for various neurological disorders.

Therefore, current research confirms that applying this technology to clinical treatment still has a long way to go. We need to develop safer, more stable, more effective, and more precise reprogramming tools and more sensitive detection and control of reprogramming techniques. The ultimate goal of reprogramming somatic cells into neurons is to obtain enough neurons to replace the neurons lost in the nervous system for various reasons. Obtaining sufficient neurons and ensuring newborn neurons correctly integrate into the local neural network after reprogramming to function appropriately for an extended period through precise and controlled reprogramming are the critical factors in applying neural reprogramming techniques in the clinical treatment of patients.

## 9. Conclusions

Somatic reprogramming has demonstrated promise in neuroregeneration and the study of neurological disorders. Small molecules and transcription factors have the ability to transform cells into neurons, which can then be used to model diseases, test drugs, and rebuild neural networks. Nonetheless, the complex environment in vivo can result in a variety of outcomes, and there is insufficient information about the safety of gene vectors in animals or humans. These are issues that must be addressed. More research is needed to improve how reprogramming for neuronal regeneration works and what it can be used for. Millions of patients around the world could benefit from more intelligent and better therapies if the many paradoxes in somatic cell reprogramming can be resolved.

## Figures and Tables

**Table 1:** Cell types that can be reprogrammed into neurons, and the neuronal subtypes after reprogramming.

Original Cell Type	Type of Reprogrammed Neuron	References
Fibroblasts	Neurons	[24]
Infrapatellar fat pad stem cells	Neurons	[34]
Astrocytes	Neurons	[26,35]
Dental stem cell	Neurons	[36]
Hematopoietic cells	Induced neuronal cells	[37]
Urine-derived cells	Induced neuronal cells	[38]
Olfactory ensheathing cells	Neuronal cells	[39]
Spermatogonial stem cells	Dopaminergic neurons	[40]
Glioma cells	Neurons	[41]
Microglia	Neurons	[42]
Striatal neurons/post-mitotic callosal neurons	Neurons	[43]
Peripheral blood T cells	Neurons	[29]
Peripheral blood mononuclear cells	Neurons	[30]
Spiral ganglion non-neuronal cells	Cochlear hair cells and cochlear nucleus neurons	[44]
Pericytes	Cholinergic neurons	[27]
Pluripotent stem cell-derived cardiomyocytes	Neurons	[45]
Oligodendrocytes	Functional neurons	[46]
NG2 cells	Neurons	[47]
Mesenchymal stem cells	Neural precursors	[48]
Hair follicle keratinocytes	Dopaminergic neurons	[32]
Müller glia	Neurogenic retinal progenitors	[49]
Adipose-derived stem cells	Neural stem cells and functional GABAergic neurons	[50]
Hepatocytes	Functional induced neuronal cells	[31]
Oligodendrocyte precursor cells	Neurons	[51]
Interfollicular keratinocytes	Neurons	[52]
Bone marrow-derived mesenchymal stem cells	GABAergic neurons	[53]

**Table 4:** List of neurodegenerative, neurological and psychiatric diseases where models based on reprogramming somatic cells into neurons have been used.

Disease	Applications/Results of Neuronal Reprogramming	Reference
Dravet syndrome	Fibroblasts derived from controls and patients were differentiated into neurons. Epilepsy-specific iPSC-derived neurons are helpful for modeling epilepsy-like hyperactivity.	[184]
MT-ATP6	Skin fibroblast reprogramming and iPSCs can model disease caused by the MT-ATP6 mutation.	[189]
Alzheimer’s disease	Small molecules induce the reprogramming of patient fibroblasts into neurons for personalized modeling of neurological disease.	[54]
Fragile X syndrome	Fibroblasts from patients can be induced into iPSC lines to enable in vitro modeling of the human disease.	[190]
Multiple sclerosis	iPSCs from peripheral blood mononuclear cells can be used to model multiple sclerosis.	[191]
Glioblastoma multiforme	Isoxazole acts as a stem cell modulator to trigger neuronal gene expression and block tumor cell proliferation, which may guide research into reprogramming as an antitumor strategy.	[41]
Frontotemporal dementia, amyotrophic lateral sclerosis	Fibroblasts were isolated from patients’ skin to generate induced pluripotent stem cells to investigate the pathological mechanisms underlying frontotemporal dementia or amyotrophic lateral sclerosis.	[192]
Huntington’s disease	Stable HD-iPS cell lines have been established to investigate disease mechanisms.	[193]
Schizophrenia	The authors directly reprogrammed fibroblasts/hair follicle-derived cells from schizophrenia patients into iPSCs, which they differentiated into neurons. These neurons were then studied for disease pathology.	[32,194]
Spinal cord injury	NOTCH1 signaling regulates the latent neurogenic program in adult reactive astrocytes after spinal cord injury.	[195]
Amyotrophic lateral sclerosis	Peripheral blood cells from an ALS patient carrying the TARDBP p.A382T mutation were reprogrammed into iPSCs.	[30]
Stroke	Overexpression of Ascl1 can convert astrocytes from the subventricular zone into neurons in vivo after stroke.	[8]
Parkinson’s disease	Fibroblasts were taken from the pathology biopsies of Parkinson’s disease patients and encouraged to develop into dopaminergic neurons, which can be used for future studies into the mechanistic underpinnings of the disease.	[151]
Rett syndrome	Overexpressing reprogramming factors in Rett syndrome fibroblasts generated iPSCs, which differentiated into neurons with a neuronal maturation phenotype similar to that of the clinical syndrome.	[196]
Neurodevelopmental disorders	Human hair follicle-derived iPSCs can be differentiated into various neural lineages. This experimental system provides an in vitro model to study normal and pathological neural development without the need for skin biopsies.	[197]
Ageing	Directly converted astrocytes retain the ageing features of the donor fibroblasts and clarify the astrocytic contribution to human CNS health and disease.	[198]
Bipolar disorder	Human fibroblasts can be reprogrammed into induced neurons or iPSCs, then differentiated into neurons for mechanistic studies of the disease.	[97]
Pain	Transcription factors can transform mouse and human fibroblasts into noxious-stimulus-detecting (injury receptor) neurons, which displayed TrpV1-mediated sensitization to inflammation.	[78]
Demyelinating diseases	Exposing mouse embryonic fibroblasts to chemical conditions could induce their differentiation into OPC-like cells, which may serve as a therapeutic strategy for treating demyelinating diseases.	[131]
Mitochondrial DNA mutations	This work generated stem cells from patients carrying the most common human disease mutation in mitochondrial DNA, m.3243A>G (MELAS).	[183]
Autism spectrum disorders	Fibroblasts from patients can be reprogrammed into neurons with fewer excitatory synapses, a faulty neural network phenotype, or a synaptic phenotype comparable to that induced by autism-associated neuroligand protein-3 mutations, confirming the use of induced neuronal cells for disease modeling.	[185,186]

Abbreviations: iPSC: induced pluripotent stem cell; MT-ATP6: mitochondrial ATP synthase subunit 6 gene; HD: Huntington’s disease; ALS: amyotrophic lateral sclerosis; TARDBP: Transactive response DNA binding protein; CNS: Central Nervous System; TrpV1: transient receptor potential vanilloid-1 channel; OPC: oligodendrocyte progenitor cells; MELAS: mitochondrial encephalomyopathy, lactic acidosis, and stroke-like episodes (MELAS) syndrome; iN: induced neuronal cells.
